# An Appendix to the Pamphlet on the Early Symptoms of Water in the Brain, Containing Cases Successfully Treated, with Practical Illustrations on the Doctrines Therein Included, and Some Observations on the Functions of the Intestines, as Connected with a Morbid Action of the Digestive Organs

**Published:** 1819-07-01

**Authors:** 


### CATALOGUE RAISONNÉE.

### I.

*An Appendix to the Pamphlet on the Early Symptoms of Water in the Brain, containing Cases successfully treated, with Practical Illustrations on the Doctrines therein included, and some Observations on the Functions of the Intestines, as connected with a Morbid Action of the Digestive Organs.* By G. D. Yeats, M. D. Fellow of the Royal College of Physicians, London, &c. 8vo. pp. 98. London, 1819.

Dr. Yeats having, in a former publication, endeavoured to impress upon the mind of the practitioner the great necessity of attending to those derangements of the abdominal functions, so commonly precursory of, and leading to, hydrencephalus, has very liberally printed his further experience in the form of an Appendix to the other work,* as an accommodation to those who purchased that publication. In this appendix there are many valuable cases, illustrative of Dr. Yeats's former doctrines and practices, and some ingenious and important reasoning on the functions of the intestines in particular. Our author justly conceives that any unusual remora or quantity of fæcal matters in the colon must prove injurious by the pressure of a hardened mass on the subjacent and circumjacent small intestines, whose functions are thereby disturbed, and whose propulsive powers are interrupted, or thrown into spasmodic action. It is easy to perceive that an overloaded and torpid state of the alimentary canal must impede the regular flow of bile and pancreatic

* A Statement of the early Symptoms which lead to the Disease termed Water in the Brain, with Observations, &c.

juice into that tube, in which event, the liver and pancreas become gorged, and morbidly affected, independently of any original fault in themselves. It is evident too, as our author observes, that a loaded state of the colon will, by impeding the propulsive powers of the intestines, diminish, or totally suspend the secretion or exhaling action on their internal surface, as is indicated by the dry, hard, and constipated state of the fæces; and by the dryness of the tongue and fauces in torpid states of the alimentary canal. This cessation of secretion from so extended a surface, must produce considerable derangement in the system at large. What secretions *are* carried on in the intestinal canal, and associated organs, during this torpid state, must also be of a morbid *quality*, as is evinced by the highly offensive smell, and bad colour of the excretions, even where very little food is taken in. It has lately been shewn that the brain is the organ which always receives an impression, whenever any part of the body is under irritation, for it is only through it that we can feel the pain to exist. "Hence it very often happens that convulsions are produced, and sometimes fatally, by local irritation in a distant part. An irritated part very commonly puts on morbid vascularity; this increased vascularity ends in an effusion of fluid, or an enlargement of the part, in the form of scirrhosity, or thickening; hence, in hydrencephalus supervening on long continued irritation of the digestive organs, we meet, not only with a watery effusion into the ventricles of the brain, but with abdominal glands morbidly enlarged; a proof, among many others, of the dangerous consequences arising from a want of proper attention to what are called general nervous feelings, or the nervous irritation of a part." 13. This we are convinced is sound reasoning, and is every day illustrated by facts, if attentive observation be paid to the phenomena of disease.

Dr. Yeats proceeds to state that, when the digestive organs of children are oppressed by retained and accumulated excretions, purgatives are usually administered; and when relief is procured, they are no more thought of till a fresh accumulation and deranged state of the system recur. But this is bad practice. "For it is not enough to have relieved the intestines of an unusual load; diseased secretions must be altered; the intestines must be gradually and healthily excited, otherwise the morbid condition soon recurs, and that dangerous irritation of the digestive organs, so much to be deprecated, supervenes." 17. Dr. Yeats reprobates the practice of giving drastic purgatives, especially of calomel, which leave a corresponding torpor of the bowels behind. Our own experience confirms Dr. Yeats's position that small alterative doses of calomel or blue pill, conjoined with such aperients as may keep up a gentle, but regular peristaltic motion in the intestines, are best suited to the class of complaints under consideration. The cases are ten in number, and are drawn up with much perspicuity and apparent fidelity, accompanied by apposite, sensible, and acute commentaries. We recommend the pamphlet to the attention of the profession at large.

